# Dual Host and Pathogen RNA-Seq Analysis Unravels Chicken Genes Potentially Involved in Resistance to Highly Pathogenic Avian Influenza Virus Infection

**DOI:** 10.3389/fimmu.2021.800188

**Published:** 2021-12-22

**Authors:** Albert Perlas, Jordi Argilaguet, Kateri Bertran, Raúl Sánchez-González, Miquel Nofrarías, Rosa Valle, Antonio Ramis, Martí Cortey, Natàlia Majó

**Affiliations:** ^1^Institut de Recerca i Tecnologia Agroalimentàries (IRTA), Centre de Recerca en Sanitat Animal (CReSA, IRTA-UAB), Campus de la Universitat Autònoma de Barcelona (UAB), Bellaterra, Spain; ^2^Departament de Sanitat i Anatomia Animals, Universitat Autònoma de Barcelona, Campus de la Universitat Autònoma de Barcelona (UAB), Bellaterra, Spain

**Keywords:** chicken, avian influenza, innate immune response, genetic resistance, RNA-Seq, PLAU

## Abstract

Highly pathogenic avian influenza viruses (HPAIVs) cause severe systemic disease and high mortality rates in chickens, leading to a huge economic impact in the poultry sector. However, some chickens are resistant to the disease. This study aimed at evaluating the mechanisms behind HPAIV disease resistance. Chickens of different breeds were challenged with H7N1 HPAIV or clade 2.3.4.4b H5N8 HPAIV, euthanized at 3 days post-inoculation (dpi), and classified as resistant or susceptible depending on the following criteria: chickens that presented i) clinical signs, ii) histopathological lesions, and iii) presence of HPAIV antigen in tissues were classified as susceptible, while chickens lacking all these criteria were classified as resistant. Once classified, we performed RNA-Seq from lung and spleen samples in order to compare the transcriptomic signatures between resistant and susceptible chickens. We identified minor transcriptomic changes in resistant chickens in contrast with huge alterations observed in susceptible chickens. Interestingly, six differentially expressed genes were downregulated in resistant birds and upregulated in susceptible birds. Some of these genes belong to the NF-kappa B and/or mitogen-activated protein kinase signaling pathways. Among these six genes, the serine protease-encoding gene *PLAU* was of particular interest, being the most significantly downregulated gene in resistant chickens. Expression levels of this protease were further validated by RT-qPCR in a larger number of experimentally infected chickens. Furthermore, HPAIV quasi-species populations were constructed using 3 dpi oral swabs. No substantial changes were found in the viral segments that interact with the innate immune response and with the host cell receptors, reinforcing the role of the immune system of the host in the clinical outcome. Altogether, our results suggest that an early inactivation of important host genes could prevent an exaggerated immune response and/or viral replication, conferring resistance to HPAIV in chickens.

## 1 Introduction

Avian influenza viruses (AIVs) are -ss RNA viruses from the family Orthomyxoviridae that can infect different avian species. They are divided into two pathotypes based on their virulence in chickens. Low pathogenic AIVs (LPAIVs) can be asymptomatic, but they typically cause mild to moderate respiratory disease, often accompanied by a decrease of water or feed consumption and drops in egg production. In contrast, highly pathogenic AIVs (HPAIVs) typically cause severe systemic disease with very high mortality in chickens (1). Due to the high mortality rates, HPAIVs represent a big economic problem to the poultry sector. Besides, AIVs are also a threat to public health, since some viral strains, e.g., viruses of the Goose/Guangdong (Gs/GD) H5 lineage, can cause human infections with more than 50% case fatality rate (2–4). The clade 2.3.4.4b H5N8 HPAIV of the Gs/GD lineage circulating in poultry and wild birds in Europe in recent years has also demonstrated its capacity to infect humans, albeit with mild clinical signs, which confirms that HPAIVs are a constant threat to public health (5, 6). The ability of an AIV to spill over to other species is linked to the evolutionary plasticity of RNA viruses that allows them to adapt to a new host faster than any other pathogen, and is often invoked to explain their zoonotic potential (7). AIVs have a segmented genome comprised of eight segments that encode 10 to 17 proteins depending on the viral strain (8). It is well known that these viruses exist in the host as quasi-species. The term viral quasi-species describes distributions of non-identical but related genomes in a cloud or swarm subjected to variation, competition and selection thought to be the target of evolutionary events (9). This is one of the reasons why RNA viruses are currently one of the highest global public health concerns (10, 11).

HPAIVs typically cause severe systemic disease, with the virus reaching different visceral organs within 24 h post-intranasal exposure and titers peaking at 48 h (12). Thus, the first hours following HPAIV infection are critical to determine survival or death, indicating the key role of the innate immune response in determining clinical outcomes and resistant phenotypes (13). Following AIV infection, host pattern recognition receptors (PRRs) recognize specific parts of the virus named pathogen-associated molecular patterns (PAMPs) to activate the innate defense. One important group of PRRs are retinoic acid-inducible gene I (RIG-I) like receptors (RLRs), composed in chickens of two cytoplasmic receptors, melanoma differentiation-associated protein 5 (MDA5) and laboratory of genetics and physiology 2 (LGP2), as highlighted in chickens by a recent RNA-Seq analysis (14). After viral recognition by the PRRs, some pathways are activated to induce the expression of type I interferon (IFN-I), which in turn enhance the expression of interferon stimulated genes (ISGs) and pro-inflammatory cytokines (15, 16). Several viral proteins are involved in the viral evasion of the innate immune response: the nucleoprotein (NP) and the matrix (M)-2 protein (M2) protein inhibit the activity of the IFN-I-dependent double-stranded RNA-activated protein kinase (PKR), which inhibits viral replication; while the non-structural (NS1) protein interferes with several innate immune signaling pathways (17).

The severity of the clinical outcome after HPAIV infection varies based on the virus strain and the host genetic background (18). Several authors have reported a different susceptibility to AIV infection depending on the chicken breed (19). Moreover, some individuals may respond differently to the infection even within the same chicken breed (20). Concerning the virus, certain AIV strains (or variants within quasi-species) are able to evade the host immune response more efficiently than others, with key amino acid mutations identified in the hemagglutinin (HA) protein (21–23). Regarding the host, polymorphisms in the *MX* gene, an ISG gene that inhibits AIV transcription and replication in mammals, have been suggested to correlate with different infection outcomes (24). However, several studies failed to demonstrate such correlation *in vivo* (20, 25, 26). An RNA sequencing (RNA-Seq) analysis between resistant and susceptible chicken breeds suggested that genes related to hemoglobin, oxygen transportation and cell adhesion played a critical role in protecting against AIV infection (27). Furthermore, genome wide association studies comparing birds that either survived or succumbed to AI outbreaks showed single nucleotide polymorphisms (SNP) in genes associated with the immune response, such as *ZNF639*, *BCL6*, or *MAPK1* (28, 29). Lack of the viral sensor RIG-I in chickens, as opposed to ducks (typically much less susceptible to HPAIV), has also been proposed as an explanation for their increased susceptibility to HPAIVs compared to ducks (16, 30). Similarly, the IFN-induced transmembrane protein (*IFITM*) gene family has been shown to be strongly upregulated in response to HPAIV in ducks compared to chickens (31). Taken together, the mechanism behind clinical outcome after HPAIV infection remains uncertain and calls for further investigation.

The outcome of HPAIV infection is a complex and multifactorial process where both the host innate immune response and the virus play an important role. The last years RNA-Seq have proved to be a powerful tool to characterize these complex processes in chickens against several pathogens (32–34). The aim of the present study was to characterize the early host and viral factors that determine the individual clinical outcome of HPAIV infected chickens. With this goal, we investigated differences in gene expression in lung and spleen in chickens that are susceptible or resistant to a HPAIV-experimentally lethal infection. Furthermore, viral quasi-species distribution was also analyzed to further understand its early contribution to infection outcome.

## 2 Materials and Methods

### 2.1 Experimental Design

Bird samples were obtained from a previous experiment conducted by our group (20). Briefly, 15-day-old chickens (*Gallus gallus*) of six different local breeds from Spain Empordanesa (EMP), Penedesenca (PENED), Catalana del Prat (C. PRAT), Flor d’Ametller (F. AMET), Castellana negra (C. NEGRA), and Euskal oiloa (E. OILOA), a commercial breed (Ross 308 Broiler), and a commercial-experimental line (specific pathogen free (SPF) White Leghorns) were used. The chickens included in this study did not receive any vaccination. Chickens were intranasally inoculated with 10^5^ mean embryo lethal dose (ELD_50;_ reciprocal of that dilution of virus per unit volume that results in the death of 50 percent of inoculated embryos) of either A/Chicken/Italy/5093/1999 (H7N1 HPAIV, Genbank accession numbers MK494920 to MK494927) or A/Goose/Spain/IA17CR02699/2017 (clade 2.3.4.4b H5N8 HPAIV, Genbank accession numbers DQ991325 to DQ991332), to assess the infection dynamics of each virus in the different breeds and to compare a recent H5N8 HPAIV isolated in Spain (Gs/GD lineage, clade 2.3.4.4b) with a classical H7N1 HPAIV. Based on our previous results, H7N1 HPAIV was more virulent to chickens than H5N8 HPAIV (20). Three chickens from each breed, including uninfected control chickens, were euthanized at 3 days post-inoculation (dpi), and a complete necropsy and tissue sampling were performed. A full set of tissues in formalin were processed for routine histopathology and immunohistochemistry (IHC) targeting the AIV NP antigen. In the present study we used lung and spleen samples collected at 3 dpi and stored at -80°C, as well as 3 dpi oral swabs.

### 2.2 Classification of Chickens as Resistant and Susceptible

In order to study expression differences relating to different host susceptibility, inoculated chickens were classified as susceptible or resistant. Chickens susceptible to infection were typified by presence of (i) evident clinical signs such as prostration, apathy and/or tremors; (ii) histopathological lesions such as hemorrhages, tissue necrosis and/or inflammation; and (iii) NP antigen in tissues associated to histopathological lesions. In contrast, chickens lacking clinical signs, histological lesions, and NP antigen in tissues at 3 dpi were classified as resistant. The breed factor was not considered for this classification since the objective was to characterize individual (not breed) factors to HPAIV resistance, as discussed later. Lung and spleen tissue samples collected at 3 dpi were used for the host transcriptome analysis and quantitative PCR validation, while 3 dpi oral swabs were used for viral quasi-species characterization (**Supplementary Tables 1A-D**). Additionally, 3 dpi oral swabs from chickens that were not necropsied were also used for the viral quasi-species analysis. In these cases, instead of the previous criteria, chickens were classified as resistant if they survived until the end of the experiment (10 dpi) with no clinical signs. In contrast, chickens that died before the end of the experiment with HPAIV compatible signs were classified as susceptible.

### 2.3 Host Transcriptome Analysis (RNA-Seq)

#### 2.3.1 RNA Preparation

Lung and spleen samples collected at 3 dpi from chickens from each group (resistant, susceptible, and control) were used. The RNA was extracted using RNeasy RNA isolation kit (Qiagen, Madrid, Spain) following manufacturer’s guidelines. The RNA was eluted in RNase free water and treated with a DNA-free kit (Ambion, Madrid, Spain). RNA quality and concentration were checked at Microomics Systems, S.L. (Barcelona, Spain) using a bioanalyzer (Agilent, Barcelona, Spain). At least three samples from lungs and spleens from control chickens and resistant and susceptible H5N8-inoculated chickens with an RNA integrity number (RIN) above eight were selected for subsequent transcriptomic analysis. However, samples from resistant H7N1-inoculated chickens showed low RNA quality and were discarded for further analysis.

#### 2.3.2 Library Preparation and Sequencing

This study initially included 36 samples from 18 chickens. The library preparation and sequencing were performed by Microomics Systems, S.L. (Barcelona, Spain). Briefly, polyadenylated mRNA molecules were purified using poly-T oligo attached magnetic beads. Following purification, the mRNA was fragmented into small pieces. The protocol involved size selection and cDNA synthesis with random primers following the Illumina TruSeq Stranded mRNA Reference Guide (35). After library preparation, sequencing of multiplexed samples was performed using Illumina Hiseq 2500 sequencer (Illumina, San Diego, CA) and 2x125 base-pair (bp) sequences were obtained with a sequencing depth by sample ranging from 20M to 37M (**Supplementary Figure 1**).

#### 2.3.3 Bioinformatics Analysis

Reads were filtered by quality control with FastQC v0.11.82 (36), then clean data were obtained by trimming adapters and nucleotides with a Phred score (a quality measure to assess the accuracy of a sequencing) of less than 30 at the beginning and end of the reads with Trimmomatic v0.39 (37). Filtered reads were mapped to the reference genome with Hisat2 v2.1.0 (38) standard settings using the galGal6 chicken assembly, and the quality control alignment was done with qualimap v.2.2.2 (39). FeatureCounts v1.6.4 (40) was used to generate table counts of each sample. Differentially expressed genes (DEGs) in samples from infected chickens compared to control chickens were normalized and obtained using the standard normalized methods performed by DESeq2 v1.24.0 (41). A principal component analysis plot of each comparison was done to detect and eliminate outliers (42). After this step, samples from one control chicken (lung and spleen), one H5N8 resistant chicken (lung and spleen), and one H7N1-susceptible chicken (lung) were discarded (**Supplementary Figure 2**). Finally, a total of 31 samples were used for further analysis. The number of samples per group analyzed were as follows: three lung samples and three spleen samples from chickens resistant to H5N8 infection; four lung samples and three spleen samples from chickens susceptible to H5N8 infection; three lung samples and four spleen samples from chickens susceptible to H7N1 infection; and five lung samples and five spleen samples from control chickens. DEGs in samples from infected chickens compared to control chickens were obtained with a Wald test and the p-value was adjusted with a Benjamini and Hochberg method (BH-adjusted p values) using DESeq2 v1.24.0 (41). DEGs with a BH-adjusted p value < 0.05 were considered significant. Heatmaps were constructed in R v4.0.5 (43) using the pheatmap package (RRID : SCR_016418). Venn diagrams were produced using the online tool Venny (https://bioinfogp.cnb.csic.es/tools/venny/index.html, accessed on 30 November 2021) To measure the viral transcript counts in each sample, the clean RNA sequencing reads were also mapped to the genome of the corresponding HPAIV strain with FeatureCounts v1.6.4 (40).

#### 2.3.4 Enrichment Analysis

Gene Ontology (GO) enrichment analysis of DEGs was performed with DAVID v6.8 (44). Most representative GO terms with a Fischer’s exact test P<0.05 after False Discovery Rate (FDR) correction were identified using REVIGO (45). A stringent dispensability cutoff (<0.05) was used, as previously described (46). KEGG pathways databases (47) were also used for a DAVID v6.8 enrichment analysis. To further elucidate if DEGs were potentially regulated by IFN-I, an enrichment analysis using Interferome v2.1 database (48) was performed.

#### 2.3.5 Validation by Quantitative PCR

To validate the results obtained by RNA-Seq, a quantitative polymerase chain reaction (qPCR) was performed for some genes of interest. RT-qPCR was performed in 18 lung samples: six samples from control chickens, and four and eight 3 dpi samples from chickens resistant and susceptible to HPAIV infection, respectively. In order to determine whether *PLAU* could be upregulated or downregulated in all the breeds used in the resistant group, at least one individual of each breed was present in the susceptible group in our qPCR validation. RNA was extracted as described above. All samples were adjusted with RNAse free water to 5 ng of RNA. A Power SYBR^®^ Green RNA-to-CT™ 1-Step Kit (ThermoFischer) was used on 7500 Fast Real-Time PCR System (ThermoFisher) to quantify gene expression levels. Primers were validated and sent by BioRad (Madrid, Spain). The housekeeping genes *ACTB*, *RPL12* and *YWHAZ* were used to normalize the results as previously described (49, 50) (**Supplementary Table 2**).

### 2.4 Viral Quasi-Species Comparison

#### 2.4.1 Sample Selection, Egg Passage Validation and Viral Quasi-Species Construction

In our previous experiment, the viral titer present at 3 dpi in oral swabs was calculated by RT-qPCR (20). The cycle threshold (Ct) values obtained (mean values around 30) suggested insufficient quantity of viral RNA to characterize the viral quasi-species using genome sequencing. Thus, in order to obtain an adequate viral titer, all 3 dpi oral swab samples were passaged in 10-day-old embryonated SPF chicken eggs by standard methods (51). To establish whether the passage in eggs introduced any viral mutations and therefore a potential bias, two oral swab samples from each virus strain with high viral titers were directly deep-sequenced before and after a 48-72 h passage. Viral quasi-species from original viral stocks and from egg allantoic fluid were inferred as previously described (52). Briefly, i) total RNA was extracted from allantoic fluid with TRIZOL LS (Ambion) and directly deep-sequenced without using primers; (ii) the genomic library was constructed for Illumina NGS using a commercial protocol and reagents (Thermo Fischer); (iii) MiSeq run of 2x250 bp was performed at Servei de Genòmica i Bioinformàtica (SGB, IBB, Barcelona); (iv) low quality reads (QC > 20) were trimmed using Trimmomatic (37); (v) quality reads were mapped against the corresponding HPAIV strain (H5N8 or H7N1) using the Burrows-Wheeler Aligner applying the BWA-MEM algorithm for long reads (53); (vi) variants were annotated with SnpSift (54) to determine the frequency of each nucleotide at each position of the reference genome; and (vii) viral quasi-species were constructed in fasta format.

In addition to the egg passage validation, the quasi-species from the two original inocula and 12 oral swabs from susceptible birds (eight H5N8-inoculated birds and four H7N1-inoculated birds) were analyzed (Ct values after the passage in eggs were <23). However, even after the passage in eggs, viral titers from resistant birds were too low for quasi-species characterization (non-detectable virus).

#### 2.4.2 Analysis of Molecular Variance (AMOVA)

To compare the viral quasi-species adaptation during the infection process, nucleotide changes between the original inocula and the 3 dpi oral swabs were identified. The average frequencies of each nucleotide per position and group (inoculum versus oral swabs for each HPAIV strain) were compared using AMOVA in Arlequin v.3.5.2.2 (55). Positions presenting fixation index values between inoculum and oral swabs larger than Fct > 0.05 were considered differentially selected, and the nucleotide change was characterized as synonymous or non-synonymous at the codon level.

## 3 Results

### 3.1 Minor Transcriptomic Changes in Tissues From Chickens That Are Resistant to HPAIV Infection

**Figure 1:**
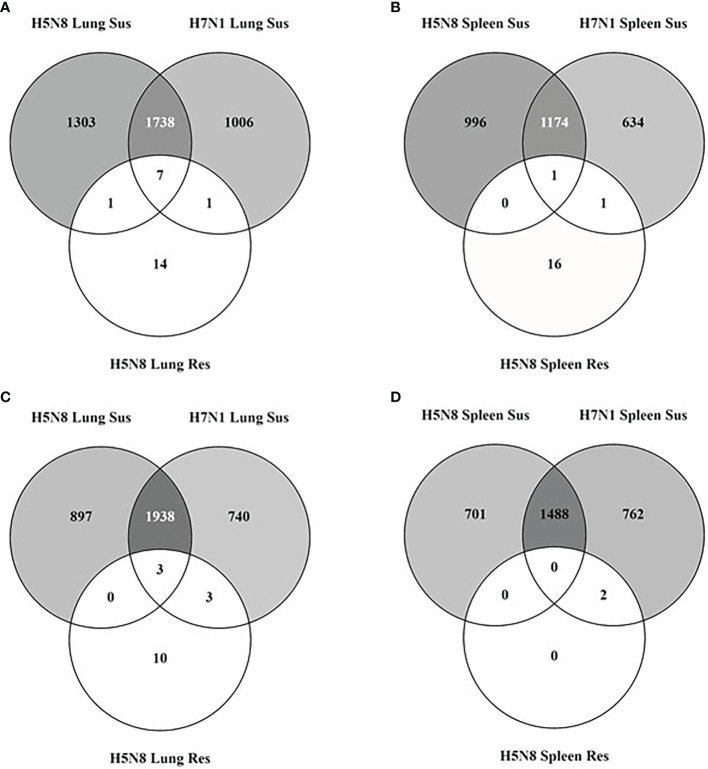
Venn diagrams illustrating overlapping of DEGs identified in lungs [upregulated **(A)** and downregulated **(C)**] and spleens [upregulated **(B)** and downregulated **(D)**] collected at 3 dpi from susceptible (Sus) and resistant (Res) H5N8 and H7N1 inoculated chickens.

Aiming to characterize the host determinants that confer resistance to HPAIV infection, we performed a transcriptomic analysis of lung and spleen tissues from resistant and susceptible chickens. Birds were infected with two different HPAIV strains (H7N1 and clade 2.3.4.4b H5N8) and samples were collected at 3 dpi, which is the earliest time point post-infection at which chickens can be categorized as resistant or susceptible by clinical signs, histopathological lesions, and IHC staining. Analysis of DEGs showed major transcriptomic changes in both lung and spleen samples from susceptible chickens compared to resistant ones (**Figure 1**). In particular, 5,887 and 5,436 DEGs from lungs, and 4,360 and 4,062 DEGs from spleens, were identified from H5N8 and H7N1 susceptible birds, respectively. More than 60% of DEGs were common in both susceptible groups in the two organs analyzed. However, more DEGs were seen in H5N8 inoculated birds than in H7N1 inoculated birds, and in lungs than in spleens. In clear contrast with susceptible chickens, only 39 and 20 DEGs were identified in lungs and spleens from resistant H5N8 inoculated birds, respectively (**Figure 1**). This marked differences validated our classification criteria, since resistant and susceptible samples clearly grouped differently in a PCA plot (**Supplementary Figure 3**). Finally, as in susceptible birds, lungs of resistant birds showed more transcriptomic changes than spleens (**Figure 1**).

**Figure 2:**
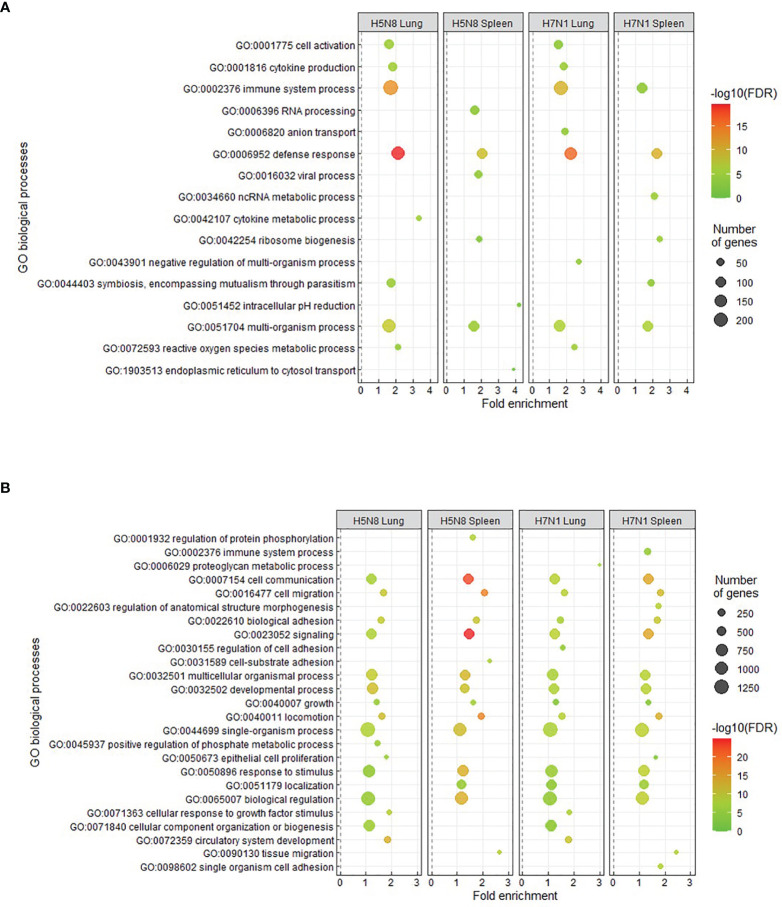
Enriched GO terms obtained from DAVID and REVIGO for upregulated **(A)** or downregulated **(B)** DEGs in lungs and spleens collected at 3 dpi from susceptible H5N8 and H7N1 inoculated chickens. The most representative and significant biological processes are represented and are sorted by fold enrichment. The dot size indicates the number of DEGs associated with the biological process. The dot color indicates the significance of the enrichment [-log10 (FDR-corrected P-values)].

Further characterization of transcriptomic changes from susceptible chickens by GO enrichment analysis showed an enrichment in processes related to immunological responses. Specifically, the most significantly upregulated GO biological processes were immune system process (GO:0002376) and defense response (GO:0006952) (**Figure 2A**). Downregulated GO biological processes were mainly related to homeostasis and growth (GO:0032502, GO:0071363) (**Figure 2B**). KEGG pathway analysis of susceptible chickens also showed enrichment in immune-related pathways such as cytokine-cytokine receptor interaction (**Supplementary Figure 4**). In general, similar enriched processes were observed for both H5N8 and H7N1 susceptible birds, but slight differences between lung and spleen were seen. More specifically, some pathways that were upregulated in lung, such as cell activation (GO:0001775), cytokine production (GO:0001816), immune system process (GO:0002376), and defense response (GO:0006952), were not differentially regulated or had a lower fold change in spleen. In contrast, enrichment analysis of DEGs from resistant birds did not generate any significant results.

**Figure 3:**
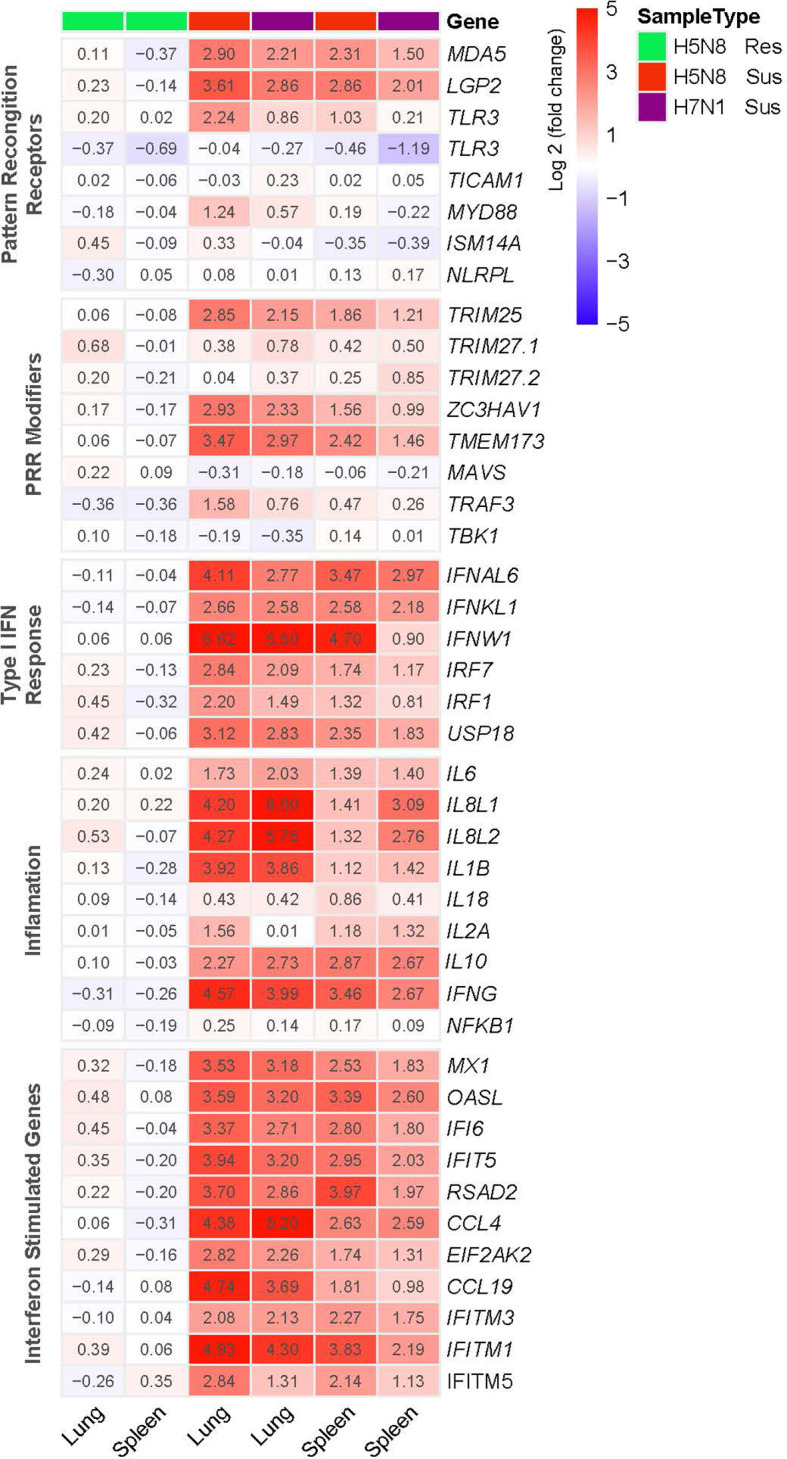
Heatmap illustrating the log2(fold change) in expression of genes representative of innate immune responses in lungs and spleens collected at 3 dpi from susceptible (Sus) and resistant (Res) H5N8 or H7N1 inoculated chickens. Columns were clustered using pheatmap (RRID : SCR_016418) in R.

To further define the transcriptional changes induced after infection, we compared the expression patterns of genes related to innate immune responses. PRR signaling as well as IFN-I and inflammatory responses were clearly upregulated in susceptible H5N8 and H7N1 inoculated birds (**Figure 3**). Besides, in line with the enrichment analysis results, susceptible H5N8 inoculated chickens had higher fold changes in these genes compared to susceptible H7N1 inoculated chickens. One exception was *IL8L1* and *IL8L2*, which showed higher expression levels in lungs and spleens of birds inoculated with the more virulent strain H7N1. Regarding differences between tissues, in line with the enrichment analysis results, lungs had higher fold changes in these genes than spleens. In contrast to susceptible birds, the expression pattern of important genes related to innate immune responses from resistant chickens lacked or presented very low differential regulation compared to the same genes from control chickens (**Figure 3**).

**Figure 4:**
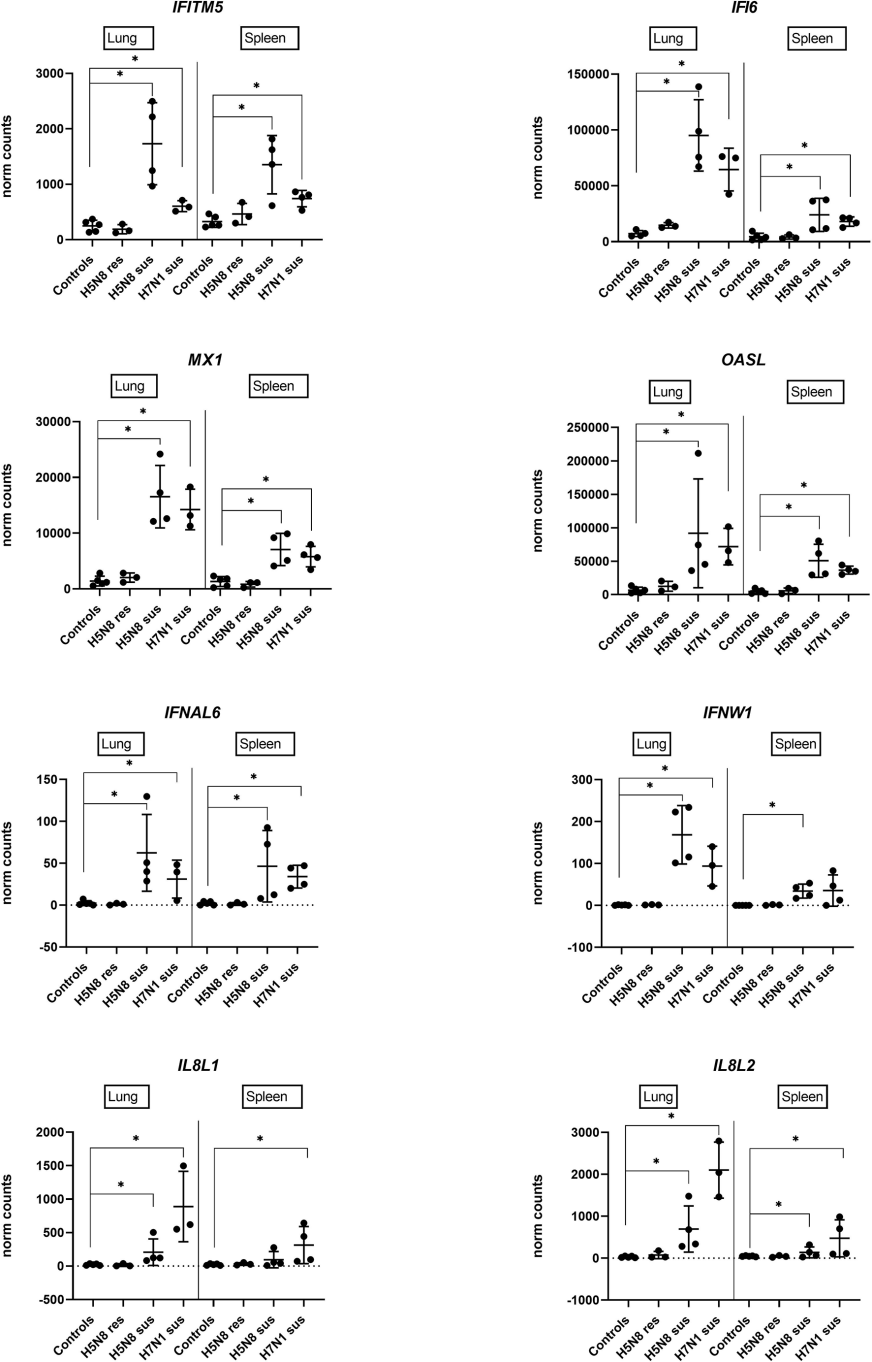
Expression levels of genes representative of IFN-I and inflammatory responses in lungs or spleens collected at 3 dpi from H5N8 or H7N1 inoculated chickens based on DEGs obtained by RNA-Seq. (∗) BH adjusted P ≤ 0.05 from Wald test p-value with DESeq2.

A deeper statistical analysis of genes representative of IFN-I and inflammatory responses (*IFITM5, IFI6, MX1, OASL, IFNAL6, IFNW1, IL8L1, IL8L2*) confirmed significantly higher expression levels in H5N8 susceptible birds and in lungs, and very low differential regulation in resistant chickens compared to controls (**Figure 4**). Furthermore, we confirmed that *IL8L1* and *IL8L2* were the only genes showing higher expression levels in susceptible H7N1 inoculated birds (**Figure 4**).

### 3.2 Low Viral Replication in Chickens That Are Resistant to HPAIV Infection

The viral transcripts from RNA-Seq data showed a clear association between the presence of virus and the number of DEGs. Higher levels of viral transcripts in lung tissues compared to spleen tissues were associated with more DEGs in lungs. Similarly, a positive correlation was also observed between viral transcripts and DEGs when comparing tissues from H5N8 and H7N1 inoculated chickens. Moreover, this analysis confirmed the lack of virus replication in lung and spleen tissues of resistant birds, which was in line with the low number of DEGs in these organs (**Supplementary Figure 5**). To further elucidate if the minor transcriptomic changes in resistant chickens were associated with lower viral replication and shedding by the oral route, we compared virus titers in 3 dpi oral swabs from resistant vs susceptible H5N8 inoculated chickens obtained in our previous experiment (20). As expected, resistant chickens had significant higher viral Ct values (Ct mean value of 35.2) compared to susceptible chickens (Ct mean value of 22.5) (**Supplementary Figure 6**).

**Figure 5:**
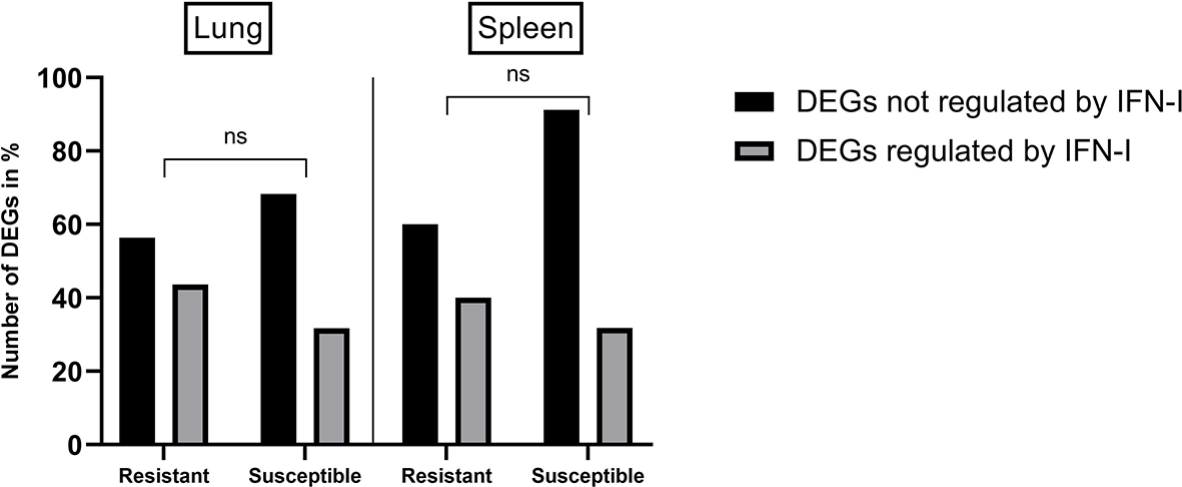
Percentages of DEGs identified in lungs and spleens from resistant and susceptible H5N8 inoculated chickens that are regulated or not by IFN-I based on results obtained from the database Interferome (v2.1). Non-significant differences in the percentages of DEGs were seen between resistant and susceptible birds (ns) P≥ 0.05; 2-way ANOVA.

To assess if an early innate immune response was induced in resistant chickens, an enrichment analysis using the Interferome v2.1 database (48) was performed. This analysis revealed that an important proportion of DEGs from lungs and spleens of H5N8 inoculated chickens are known to be regulated by IFN-I, in even higher percentages than the ones observed in susceptible birds (**Figure 5**).

### 3.3 Downregulation of the Serine Protease Encoding Gene *PLAU* in Lungs Correlates With Resistance to HPAIV Infection

**Figure 6:**
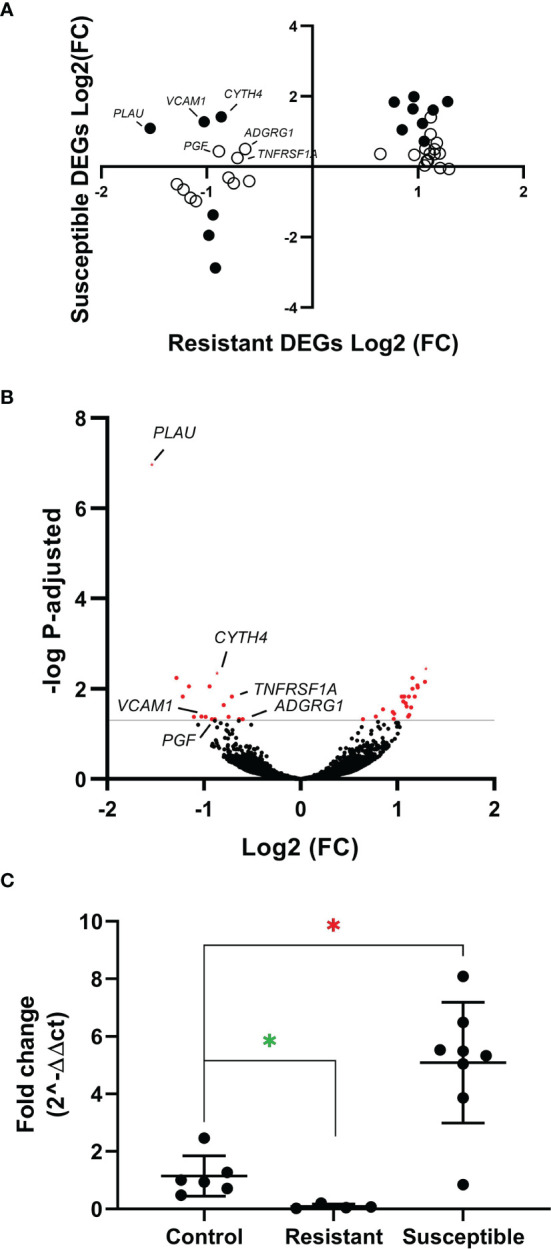
**(A)** Expression pattern of the 39 resistant-specific DEGs in lungs from susceptible (y-axis) and resistant (x-axis) chickens. Fold changes of the 39 genes in both groups are represented. Black dots indicate DEGs in both susceptible and resistant birds. White dots indicate DEGs only in resistant birds. **(B)** Volcano plot showing all genes identified by RNAseq in lungs from resistant chickens. In red are shown the 39 DEGs (BH adjusted P < 0.05). **(C)** Quantitative PCR of *PLAU* from lungs of resistant and susceptible H5N8 inoculated chickens collected at 3 dpi. Lungs from uninfected chickens were used as control. For each group the mean and standard deviation are shown. Green asterisk means significant downregulation and red asterisk means significant upregulation (∗) BH adjusted P ≤ 0.05 from Wald test p-value with DESeq2.

**Figure 7:**
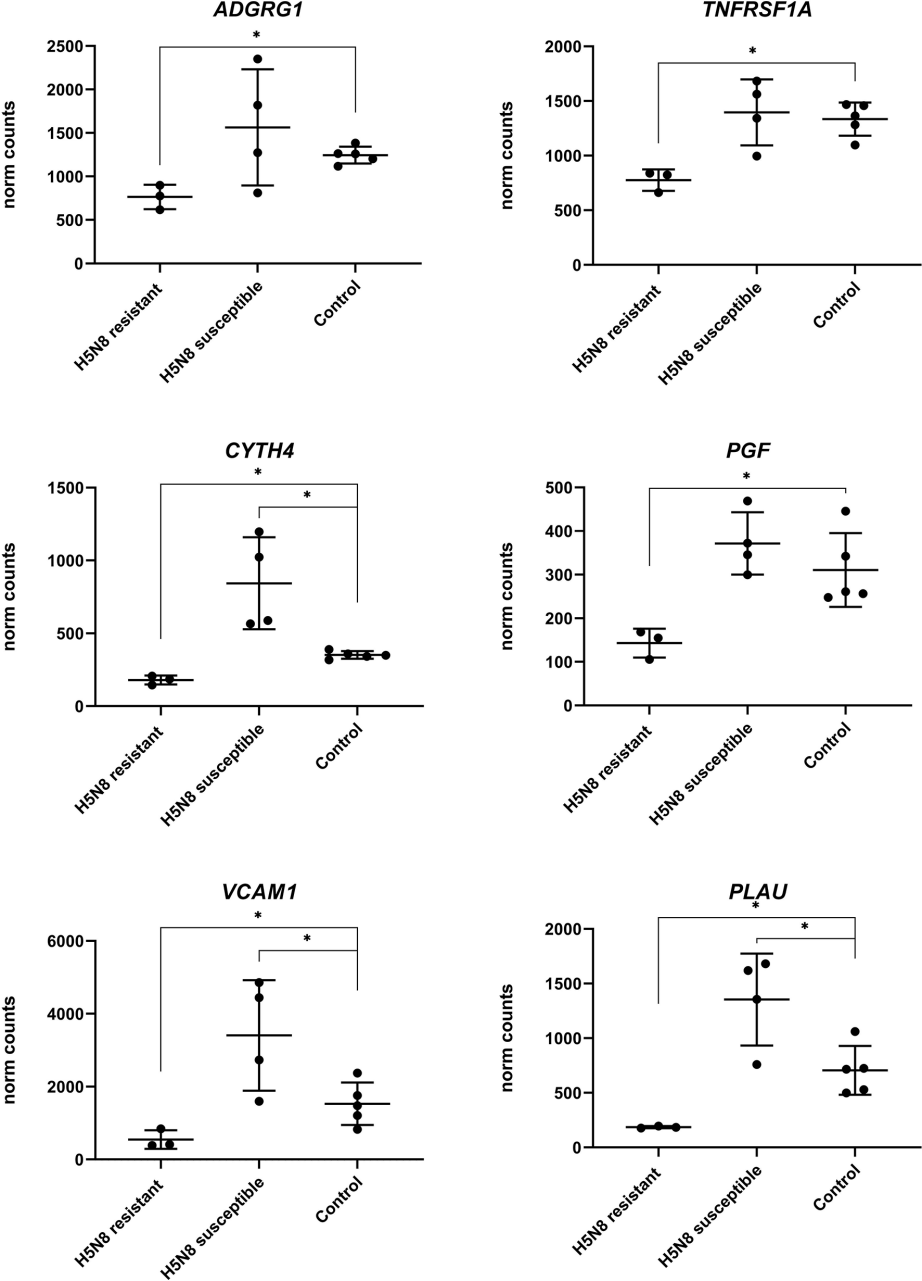
Expression levels of the six genes with opposite regulation between resistant and susceptible chickens inoculated with H5N8 virus from lungs collected at 3 dpi. For each group the mean and standard deviation are shown. (∗) BH adjusted P ≤ 0.05 from Wald test p-value with DESeq2.

To further characterize the minor transcriptomic changes observed in resistant chickens, we grouped the 39 DEGs identified in lungs from resistant H5N8 inoculated birds, compared to control birds, by their expression patterns (**Supplementary Table 3**). Most of the 39 genes were similarly upregulated or downregulated in both resistant and susceptible birds (**Figure 6A**). Among them, we found genes related to ion homeostasis (*SLC24A4, EPB42, RHAG*), immune system process (*BLOC1S6, FGF14, EPB42, ANKRD54, RHAG*), and response to stimulus (*DGKG, BLOC1S6, PRLHRL, FGF14, RRAD, ANKRD54*). Interestingly, six DEGs followed a different expression pattern, being downregulated only in resistant birds and upregulated in susceptible birds (**Figures 6A**, **7**). Some of these genes belong to the NF-kappa B (NF-κB) (*PLAU, VCAM1, TNFRSF1A*) and/or mitogen-activated protein kinase (MAPK) (*TNFRSF1A* and *PGF*) signaling pathways (47), indicating a differential regulation of the inflammatory response in lungs for both infection outcomes. Among these six genes, the serine protease-encoding gene *PLAU* was of particular interest, being the most significantly downregulated in resistant chickens (**Figure 6B** and **Supplementary Table 4**). Moreover, qPCR analysis of *PLAU* in a higher number of resistant chickens (**Supplementary Table 1C**) showed a statistically significant downregulation when compared not only to susceptible chickens (which upregulated this gene), but also compared to controls, thus further validating a differential response between resistant and control chickens (**Figure 6C**). Overall, these results demonstrate a differential host response against HPAIV in successfully inoculated resistant and susceptible chickens, always in contrast with control chickens

### 3.4 AMOVA Results Point to Few Quasi-Species Differences Between Inocula and 3 dpi Oral Swabs of Susceptible H5N8 or H7N1 Inoculated Chickens

In order to validate the egg passage technique, the nucleotide frequencies per position were compared between four non-passaged samples (two H5N8 samples and two H7N1 samples) and the same samples after 48-72 h of egg-passage in SPF embryonated eggs. The comparison of the quasi-species obtained per segment with both treatments indicated a bias in the mean nucleotide frequencies per position that ranged between 0.5% in the neuraminidase (NA) of H7N1 to 1.2% in the polymerase basic 1 (PB1) of H5N8, with most values around 0.7%. All nucleotide frequencies were below the fixation index value (5%) selected for a nucleotide change to be considered significant.

The comparison of the nucleotide frequencies per segment between the original inocula and the 3 dpi oral swabs of susceptible birds offered contrasting results (**Supplementary Figure 7**). Regarding H5N8 virus, no significant differences between the original H5N8 inoculum and the oral swabs of susceptible H5N8 inoculated chickens were detected in the HA, M, NP, polymerase acidic (PA), and PB2 segments, and only PB1 (n=7), NA (n=1), and non-structural protein (NS) (n=1) showed significant differences. The seven PB1 changes were located between nucleotide positions 246-346 (amino acid residues 82-115), a region known to have an alternative codification for PB1-F2 (nucleotides 227-385 of the PB1 A/goose/Spain/IA17CR02699/2017 (H5N8), MK494921). All nucleotide changes codified for non-synonymous mutations in the PB1 and PB1-F2 proteins. Regarding H7N1 virus, significant Fct values between the original H7N1 inoculum and oral swabs of susceptible H7N1 inoculated chickens were observed in 91 nucleotide positions, most of them in the PA (40 nucleotides, 37 non-synonymous) and PB2 (45 nucleotides, 43 non-synonymous) segments. These mutations were located between amino acid residues 572-656 of PA and 60-137 of PB2. Few nucleotide differences were detected in HA (n=2), M (n=1), and NP (n=1), while no differences were reported in NA and NS (**Supplementary Table 5**).

## 4 Discussion

For an infection event to be successful, a pathogen must overcome the physical and immunological barriers of the host. Focusing on the pathogen of interest here, two HPAIV strains (H7N1 and clade 2.3.4.4b H5N8) were experimentally inoculated in a diverse group of chicken breeds (20). Here, the transcriptome of resistant and susceptible birds (classified by clinical signs, lesions, and presence of viral antigen in tissues) and the viral quasi-species after infection were characterized. The results obtained showed the differences that are linked to host susceptibility to virus infection.

In our previous study, H7N1 HPAIV strain was more virulent than H5N8 HPAIV strain in different chicken breeds since it caused the highest frequency of severe clinical signs, highest mortality rates (70% versus 47% mortality rate), and shortest mean death time (3.3 dpi versus 4.9 dpi) (20). Nonetheless, transcriptomic results in the present study indicated that both virus strains generally induced similar host transcriptional responses in susceptible birds. More specifically, a similar expression level was observed in IFN-I response, cytokine production, and ISG, even though susceptible H5N8 inoculated chickens presented more DEGs and slightly higher fold changes in immune-related DEGs, probably associated to a higher viral replication rate in lung and spleen of H5N8 inoculated chickens compared to H7N1 inoculated chickens (20). However, a stronger *IL8L1* and *IL8L2* chemokine response was detected in lung and spleen of H7N1 inoculated chickens, perhaps a biomarker of an aberrant innate immune response (56) as seen before with this virus strain (57). It is worth mentioning that differences in clinical outcome between the two HPAIV strains are mainly due to higher viral replication in the heart, central nervous system, and pancreas of H7N1 inoculated birds as previously discussed (20). A similar gene expression was also observed between tissues (lung and spleen) of susceptible birds. Nevertheless, lung samples presented more DEGs, GO enriched pathways, and higher fold changes than spleen samples. These differences, as well as those observed when comparing the two virus strains, can be attributed to a different viral replication rate between tissues, since higher viral titers were detected in lungs compared to spleens. Furthermore, because trypsin‐like enzymes in respiratory epithelial cells can cleave the viral HA and allow entry of the virus, multiple replication cycles occur in the lung (18). Altogether, these factors could explain why more virus and, consequently, more DEGs, were detected in lungs compared to spleens.

After considering the transcriptomic differences in susceptible birds between HPAIV strains and tissues, we focused on the differences between resistant and susceptible H5N8 inoculated chickens. The experimental design did not take into consideration the influence of the different chicken breed in the susceptibility of the infection, since we had previously shown that both susceptible and resistant chickens are present in different breeds (20). We thus hypothesized that all chickens resistant to infection share a conserved transcriptomic signature in response to viral exposure regardless of their breed. This approach based on the comparison of individual chickens with different infection outcomes makes our analysis unique, in contrast with similar recent chicken RNA-Seq studies where resistant and susceptible chicken lines are compared instead (33, 34, 58). A salient feature were the minor transcriptomic changes observed in resistant chickens versus controls, in contrast with the major transcriptomic changes in susceptible chickens. Based on these results, the pressing question was whether resistant chickens were actually infected, since they lacked direct evidence of IFN-I or inflammatory responses. Nevertheless, three main reasons support the fact that resistant chickens were infected; 1) the high viral dose used for the experimental infections (59); 2) the fact that within the 39 DEGs obtained in resistant chickens in contrast to control chickens, we found IFN-I regulated genes (Interferome v2.1 database) and genes related to defense response (Gene Ontology analysis); and 3) some DEGs downregulated in resistant chickens but upregulated in the susceptible ones were related to innate immune pathways such as Nf-kB and MAPK signaling. However, our study offers a static picture of the HPAIV infection at 3 dpi, which permits the comparison of resistant and susceptible birds based on clinical signs, lesions, and presence of antigen. It will be interesting to further characterize this resistant-specific responses analyzing other relevant tissues and time-points. Indeed, the few differentially expressed genes identified in these chickens might indicate that a more robust host response may occur in the upper respiratory tract or at earlier time-points post-infection. Given that AIV pathogenesis in chickens is determined by early immune responses (60–63), these minor transcriptomic changes in lungs could be associated to a concomitant innate immune response in nasal mucosa where the virus first replicates and finds the first host barrier (64). Finally, our study had a limitation in the number of resistant chickens analyzed, which probably resulted in the low number of DEGs observed. Despite this fact does not play down the results obtained, it is possible that analyzing a higher number of samples would unmask a broader transcriptomic response in lungs.

Despite most of the few DEGs from resistant chickens showed similar expression patterns to those in susceptible chickens, six of these DEGs showed an opposite sense of regulation. All six of these genes were found in lungs and were downregulated in resistant chickens and upregulated in susceptible ones. Three of the downregulated genes (*PLAU, VCAM1, TNFRSF1A*) belong to the NF-κB signaling pathway, suggesting that the activation of this inflammatory pathway might benefit virus replication. In fact, some drugs inhibit this pathway as a strategy against influenza viruses (65–67). However, this host signaling pathway seems to play a controversial role in AIV infection. Even if low levels of NF-κB activity allow cells to avoid viral replication by retaining newly synthesized viral ribonucleoprotein complexes (vRNP) in the nucleus (68–70), complete inhibition of NF-κB functions can increase viral replication (71). Apart from the NF-κB pathway, two genes (*TNFRSF1, PGF*) downregulated in resistant chickens and upregulated in susceptible ones are related to MAPK pathway. As in the case of NF-κB, the inactivation of the classical MAPK cascade leads to reduced viral titers by the retention of vRNP in the nucleus (72, 73). Altogether, these results suggest an early differential regulation of intracellular signaling pathways between resistant and susceptible chickens.

Moreover, the *TNFRSF1A* gene, which is involved in the influenza A infection pathway (47), is a tumor necrosis factor (TNF)-alpha receptor that recruits caspase-8; its activation initiates the subsequent cascade of caspases (aspartate-specific cysteine proteases) that mediates apoptosis (74, 75). Downregulation of *TNFRSF1A* could play a role in the retention of vRNP avoiding a caspase-mediated disruption of the nuclear pore complex and a subsequent reduction of viral replication, as discussed above. Interestingly, the gene *PGF*, a growth factor which is active in angiogenesis and endothelial cell growth (75), enhances the magnitude and duration of TNF-alpha and toll-like receptors pathways, contributing to the subsequent release of inflammatory cytokines (76–78). Thus, downregulation of *PGF* in resistant birds could avoid an exaggerated pathologic pro-inflammation in response to viral infection. Finally, the gene *VCAM1* has cell adhesion functions, more particularly in leukocyte-endothelial cell adhesion (79). Its downregulation could suggest a resolution of a previous inflammatory process, reinforcing the hypothesis of an early immune response controlled during the very first hours.

Finally, the DEG found in lungs from resistant chickens showing the highest downregulation was *PLAU* (also known as *UPA*), which in contrast was clearly upregulated in susceptible birds, suggesting a key role in the final outcome of the disease. Its differential expression was further validated by qPCR from a larger number of samples of resistant, susceptible and control chickens from the same experiment. Additionally, in order to determine whether *PLAU* gene could be upregulated or downregulated in all the breeds used in the resistant group, at least one individual of each breed was present in the susceptible group in our qPCR validation. Our results indicate that different individuals of the same breed were able to regulate differentially this gene. Further studies are required in order to elucidate if *PLAU* expression is differentially regulated in different chicken breeds, through divergent epigenetic mechanisms or other transcriptional regulatory factors. *PLAU* encodes a serine protease that converts plasminogen to plasmin (79) and plays a relevant role in vascular degradation by increasing VE-cadherin degradation and in inflammation (80–82). Our main hypothesis was related with the effect of *PLAU* in the plasminogen conversion to plasmin. This could be relevant in the context of HPAIV infection in two different ways; avoiding viral replication and/or avoiding fibrinolysis (83). Specifically, plasmin is a serine-protease known to participate in the cleavage of the HA protein into HA1 and HA2, a necessary step for AIV infectivity (84, 85) Thus, the downregulation of *PLAU* in resistant birds could lead to an inefficient AIV replication in the lung. Furthermore, the conversion of plasminogen to plasmin by *PLAU* is related to fibrinolysis-mediated inflammation (86, 87), which has been suggested to play a role in a deleterious lung inflammation by increasing fibrin degradation products and vascular permeability in a mouse model (83). We hypothesize that an optimized regulation of *PLAU* could reduce the coagulation dysfunction that HPAIV typically produce (88). Besides, *PLAU* is also known to play a relevant role in other viral infections, such as a biomarker of coronavirus disease (COVID-19) severity (89, 90), associated with diffuse hemorrhages in classical swine fever virus (91), and with viral fusion to the cellular membrane in human immunodeficiency virus 1 infection (92). Altogether, these results suggest that the downregulation of *PLAU* could have a relevant impact in the outcome of HPAIV disease in resistant chickens by avoiding vascular damage and permeability, deleterious lung inflammation and/or viral activation.

From the virus side, the AMOVA analyses identified several positions with significant changes along the viral quasi-species of 3 dpi oral swabs of susceptible H5N8 inoculated birds, mostly in PB1 (seven out of nine), whereas a large proportion of changes identified in H7N1 inoculated birds were located in PA and PB2. All three segments constitute the polymerase heterotrimer of AIV, which is involved both in transcription and replication of the viral RNA genome (93). The PB1 protein is in the core of this complex, with its N and C terminal domains directly interacting with the C terminal domain of PA and the N terminal domain of PB2, respectively (94). The mutations detected in PB1 are located just downstream of this interaction stretch, before the ATP binding site, in a region that encodes for PB1 and PB1-F2 proteins. Considering that all seven nucleotide mutations in PB1 and PB1-F2 of H5N8 inoculated birds were non-synonymous, those changes could imply a fitness gain for the virus, although further studies are needed to confirm this hypothesis. Most mutations observed in H7N1 inoculated birds were non-synonymous changes in the C terminal domain of PA and in the N-terminal domain of PB2, which is known to interact with PB1. Also, most mutations introduced amino acids with lower, more acidic, isoelectric points, potentially offering a fitness win. The H5N8 HPAIV strain used in this study is a goose-origin isolate and is less adapted to chickens than the H7N1 HPAIV strain that is a chicken-origin isolate (20). This should have implied fewer changes along the viral quasi-species from H7N1 inoculated birds, as seen in more adapted strains (95), but the opposite was observed. This observation could be explained by the particularities of the randomly chosen samples analyzed, since oral swabs from H5N8 inoculated chickens had a higher replication rate than oral swabs from H7N1 inoculated chickens, offering a skewed picture of viral evolution dynamics. Interestingly, we found no or few substantial changes in the segments that encode for the proteins that interact with the innate immune system (NP, NS, and M) or the cell receptors (HA and NA), indicating no adaptation in the segments that interact with the innate immune system of susceptible birds during the first 3 dpi. Regrettably, the viral quasi-species of resistant birds could not be analyzed, as they quickly cleared the infection. However, because of their shorter viral shedding, nucleotide differences in quasi-species of resistant birds are unlikely. In susceptible birds, the lack of virus variation in the segments known to interact with the innate immune system of the host, coupled with the striking differences in gene regulation between susceptible and resistant birds, points towards a scenario where successful infection events seem not to be due to the changes in the viral quasi-species, but to the ability of the host’s immune system to mount an early and efficient response against the pathogen.

To sum up, some immune-related pathways and genes that could play a role in the outcome of the disease caused by HPAIV in chickens were identified. An early and optimal regulation of the NF-κB pathway and the MAPK cascades could be the reason why some chickens are resistant to AIV. This optimal host regulation could avoid an exaggerated immune response and/or viral replication by an early inactivation of important genes like *PLAU*. The relevance of the transcriptomic results is strengthened by the viral quasi-species analyses, where no evidence of viral adaptation to the host was reported among susceptible birds in the segments known to interact with the innate immune system. Although our results strongly suggest the implications of specific genes in HPAIV clinical outcome, further research is needed evaluating upper respiratory tissues at earlier time-points after exposure in order to confirm their contribution.

## Data Availability Statement

The datasets presented in this study can be found in online repositories. The names of the repository/repositories and accession number(s) can be found below: https://www.ebi.ac.uk/metagenomics/, PRJEB48271.

## Ethics Statement

The experimental design was approved by the ethical Committee of Institut de Recerca i Tecnologia Agroalimentàries (IRTA) and the Government of Catalonia (Departament de Territori i Sostenibilitat, Direcció General de Polítiques Ambientals i Medi Natural) under reference code CEEA 18/2017-9457.

## Author Contributions

The project was conceived, and funding obtained by MN, AR, and NM. The experiment was designed by RS-G, AP, RV, and NM. The experiment was performed by RS-G and samples collected and processed by AP and RV. JA provided assistance with the analysis of RNA-Seq data. MC provided assistance with the quasi-species analysis. Figures were prepared by JA, AP, MC, and KB. The manuscript was drafted by AP with corrections from MC, JA, KB, and NM. All authors contributed to the article and approved the submitted version.

## Funding

This work was supported by the coordinated project RTA2015- 00088-C03-03 of the Instituto Nacional de Investigación y Tecnología Agraria y Alimentaria (INIA) and Spanish Ministerio de Ciencia Innovación, by the coordinated project PID2020-114060RR-C33-INFLUOMA, to authors NM and AR.

## Conflict of Interest

The authors declare that the research was conducted in the absence of any commercial or financial relationships that could be construed as a potential conflict of interest.

## Publisher’s Note

All claims expressed in this article are solely those of the authors and do not necessarily represent those of their affiliated organizations, or those of the publisher, the editors and the reviewers. Any product that may be evaluated in this article, or claim that may be made by its manufacturer, is not guaranteed or endorsed by the publisher.
