# Corrigendum: Dual Host and Pathogen RNA-Seq Analysis Unravels Chicken Genes Potentially Involved in Resistance to Highly Pathogenic Avian Influenza Virus Infection

**DOI:** 10.3389/fimmu.2022.939849

**Published:** 2022-05-30

**Authors:** Albert Perlas, Jordi Argilaguet, Kateri Bertran, Raúl Sánchez-González, Miquel Nofrarías, Rosa Valle, Antonio Ramis, Martí Cortey, Natàlia Majó

**Affiliations:** ^1^Institut de Recerca i Tecnologia Agroalimentàries (IRTA), Centre de Recerca en Sanitat Animal (CReSA, IRTA-UAB), Campus de la Universitat Autònoma de Barcelona (UAB), Bellaterra, Spain; ^2^Departament de Sanitat i Anatomia Animals, Universitat Autònoma de Barcelona, Campus de la Universitat Autònoma de Barcelona (UAB), Bellaterra, Spain

**Keywords:** chicken, avian influenza, innate immune response, genetic resistance, RNA-Seq, PLAU

In the original article, we neglected to include the funder **Spanish Ministerio de Ciencia e Innovación**, by the coordinated project **PID2020-114060RR-C33-INFLUOMA**, entitled “*Unravelling the molecular mechanisms of avian influenza virus infection outcome in the avian host by using a multi-omics approach*” to authors **Natàlia Majó** and **Antonio Ramis**.

The authors apologize for this error and state that this does not change the scientific conclusions of the article in any way. The original article has been updated.

## Publisher’s Note

All claims expressed in this article are solely those of the authors and do not necessarily represent those of their affiliated organizations, or those of the publisher, the editors and the reviewers. Any product that may be evaluated in this article, or claim that may be made by its manufacturer, is not guaranteed or endorsed by the publisher.

